# Effects of semaglutide on risk of cardiovascular events across a continuum of cardiovascular risk: combined post hoc analysis of the SUSTAIN and PIONEER trials

**DOI:** 10.1186/s12933-020-01106-4

**Published:** 2020-09-30

**Authors:** Mansoor Husain, Stephen C. Bain, Anders Gaarsdal Holst, Thomas Mark, Søren Rasmussen, Ildiko Lingvay

**Affiliations:** 1 Ted Rogers Centre for Heart Research, Department of Medicine, University of Toronto, Toronto, ON Canada; 2 Swansea University Medical School, Swansea, UK; 3 Novo Nordisk A/S, Søborg, Denmark; 4 University of Texas Southwestern Medical Center, Dallas, TX USA

**Keywords:** Type 2 diabetes, Subcutaneous semaglutide, Oral semaglutide, Cardiovascular, MACE, SUSTAIN, PIONEER, Risk prediction model

## Abstract

**Background:**

Semaglutide is a glucagon-like peptide-1 (GLP-1) analog treatment for type 2 diabetes (T2D) available in subcutaneous (s.c.) and oral formulations. Two cardiovascular (CV) outcomes trials showed that in subjects with T2D at high risk of CV events there were fewer major adverse CV events (MACE; defined as CV death, non-fatal stroke, non-fatal myocardial infarction) with semaglutide than with placebo (hazard ratio [95% CI]: 0.74 [0.58;0.95] for once-weekly s.c. semaglutide and 0.79 [0.57;1.11] for once-daily oral semaglutide). However, there is little evidence for an effect of semaglutide on MACE in subjects not at high risk of CV events. This post hoc analysis examined CV effects of semaglutide in subjects across a continuum of baseline CV risk.

**Methods:**

Data from the s.c. (SUSTAIN) and oral (PIONEER) semaglutide phase 3a clinical trial programs were combined according to randomized treatment (semaglutide or comparators) and analyzed to assess time to first MACE and its individual components. A CV risk model was developed with independent data from the LEADER trial (liraglutide vs placebo), considering baseline variables common to all datasets. Semaglutide data were analyzed to assess effects of treatment as a function of CV risk predicted using the CV risk prediction model.

**Results:**

The CV risk prediction model performed satisfactorily when applied to the semaglutide data set (area under the curve: 0.77). There was a reduced relative and absolute risk of MACE for semaglutide vs comparators across the entire continuum of CV risk. While the relative risk reduction tended to be largest with low CV risk score, the largest absolute risk reduction was for intermediate to high CV risk score. Similar results were seen for relative risk reduction of the individual MACE components and also when only placebo comparator data were included.

**Conclusion:**

Semaglutide reduced the risk of MACE vs comparators across the continuum of baseline CV risk in a broad T2D population.

*Trial registrations* ClinicalTrials.gov identifiers: NCT02054897, NCT01930188, NCT01885208, NCT02128932, NCT02305381, NCT01720446, NCT02207374, NCT02254291, NCT02906930, NCT02863328, NCT02607865, NCT02863419, NCT02827708, NCT02692716, NCT02849080, NCT03021187, NCT03018028, NCT03015220.

## Background

Semaglutide (Novo Nordisk, Denmark) is a glucagon-like peptide-1 (GLP-1) analog for the treatment of type 2 diabetes (T2D) [1, 2]. There are two formulations of semaglutide: once-weekly subcutaneous (s.c.) semaglutide and once-daily oral semaglutide. Although the route of administration differs between the two semaglutide formulations, they share similar pharmacokinetic profiles and clinical effects once they have been absorbed into the bloodstream [3–7].

Both formulations were extensively studied in clinical trial programs: s.c. semaglutide in nine SUSTAIN phase 3a clinical trials (SUSTAIN 1–6, two Japanese trials and the China Multi-Regional Clinical Trial) [8–16] and four phase 3b clinical trials (SUSTAIN 7–10) [17–20] and oral semaglutide in the 10 PIONEER phase 3a clinical trials (PIONEER 1–10; PIONEER 9 and 10 were conducted in Japan) [21–30] to date. Both clinical trial programs included cardiovascular outcomes trials (CVOTs): SUSTAIN 6 (s.c. semaglutide) and PIONEER 6 (oral semaglutide) [13, 26]. In these trials, which had similar designs and included populations enriched for subjects at high risk for cardiovascular (CV) events [13, 26], there were fewer major adverse CV events (MACE, defined as death from CV causes, non-fatal myocardial infarction [MI] or non-fatal stroke) with semaglutide vs placebo: the hazard ratios were similar for s.c. semaglutide (0.74 [95% confidence interval (CI) 0.58;0.95]) and oral semaglutide (0.79 [95% CI 0.57;1.11]), with the former being statistically significant [13, 26].

The aim of this analysis was to better understand the CV effects of semaglutide in terms of both relative and absolute risk reduction in the broader range of T2D patient profiles that are encountered in routine clinical practice. To achieve this, we estimated CV treatment effects by conducting a post hoc meta-analysis of data from SUSTAIN and PIONEER phase 3a trials, with subjects distributed across the continuum of baseline CV risk. Subjects were distributed using a CV risk prediction model developed using an independent dataset from the LEADER CVOT, which evaluated CV outcomes with liraglutide vs placebo. LEADER had the same endpoint definitions and explanatory variables as the SUSTAIN and PIONEER clinical trial programs [31].

## Methods

### Combined semaglutide trial data

This analysis used baseline and adjudicated CV outcomes data from the 18 phase 3a SUSTAIN and PIONEER trials published at the time of writing: SUSTAIN 1–6, two SUSTAIN Japanese trials and PIONEER 1–10. Data were pooled according to the randomized treatment; semaglutide (0.5 and 1.0 mg s.c. and 3, 7 and 14 mg oral) or comparator (placebo, sitagliptin, exenatide extended release, insulin glargine, dulaglutide, liraglutide and empagliflozin). This pooling was appropriate because the same definitions for baseline variables and MACE, which were ensured by a consistent event adjudication process, were used across the trials.

Trial information (including aims, designs and interventions) can be found in the publications of the individual trials [8–15, 21–30].

### Statistical methods

#### CV risk prediction model

As described previously, an identical 3-point composite MACE endpoint was the primary outcome for the LEADER, SUSTAIN 6 and PIONEER 6 CVOTs, and was defined as time to first occurrence of death from CV disease (CVD, including undetermined causes), non-fatal MI or non-fatal stroke [13, 26, 31]. The same endpoint was available for the glycemic efficacy trials (SUSTAIN 1–5, the two SUSTAIN Japanese trials, and PIONEER 1–5 and 7–10).

Data from the LEADER CVOT [31] were used to develop a CV risk score to distribute subjects according to CV risk. To identify baseline characteristics for inclusion in the CV risk prediction model, a set of characteristics known to be important (or potentially important) predictors of CV risk, common to both LEADER and the pooled semaglutide randomized controlled trials (RCTs), were considered in a Cox proportional hazard model with stepwise selection using SAS PHREG (SAS, 2019); p-values  < 0.05 determined whether a predictor should be included in or removed from the model. The baseline variables selected for use in the model are listed in Additional file 1: Appendix Table S1.

Using the estimated coefficients of the baseline characteristics that remained in the CV risk prediction model, a risk score could then be calculated for LEADER and extrapolated to other trials that measured the same baseline characteristics. A lower risk score from the model indicates lower CV risk at baseline and, conversely, a higher risk score indicates higher CV risk at baseline.

The Harrell’s C-statistic (area under the curve [AUC]) was used to evaluate the applicability of the CV risk prediction model to the semaglutide data (overall, efficacy trials alone, CVOTs alone), with values of 1.0 indicating a perfect predictive performance, 0.9–1.0 indicating an excellent performance, 0.8–0.9 indicating a good performance and 0.7–0.8 indicating a fair performance [32]. Absolute risk plots were also used to evaluate the validity of the model.

Of note, only the first two years of observation time from LEADER were considered in the model selection to align with the durations of the trials included in the SUSTAIN and PIONEER programs. An overall summary of the LEADER risk score model, including the interactions considered in it, are outlined in Additional file 1: Appendix Table S1.

#### Time to first MACE (semaglutide vs comparators) across the continuum of baseline CV risk

For the main endpoint (time to first MACE), and for each individual component of MACE, the combined semaglutide trial data were analyzed for up to 2 years (to align with the observation time used as the basis for the risk prediction model) using a Cox proportional hazards model stratified according to a 4-group categorization by trial program (SUSTAIN or PIONEER) and trial type (CVOT or glycemic efficacy trial).

The explanatory variables in the model were randomized treatment (semaglutide or comparator), baseline CV risk score (from the CV risk prediction model derived from the LEADER data as described above) and the interaction between treatment and CV risk score.

Individual subject profiles are presented according to low (5th percentile), midpoint or high (95th percentile) CV risk, to illustrate an example of the baseline characteristics associated with each CV risk classification. Numbers needed to treat (NNT) were also calculated as 1/absolute risk reduction.

Further details on the semaglutide model selection (including Akaike Information and Schwarz Bayesian Information Criteria) can be found in Additional file 2: Appendix Table S2. Generally, models within a difference of < 2 compared with the model with the lowest value in relation to the specific information criteria can be considered similar in a goodness-of-fit model adjusted for the number of parameters it includes [33, 34].

## Results

### Baseline characteristics

Pooled subject numbers and baseline characteristics for the semaglutide RCTs are summarized in Table 1. At baseline, age and the proportion of subjects with heart failure, prior ischemic heart disease, prior MI, prior stroke and who used insulin were higher in the CVOTs than in the pooled glycemic efficacy trials, while low-density lipoprotein (LDL)-cholesterol, estimated glomerular filtration rate (eGFR) and the proportion of current smokers were higher in the glycemic efficacy trials than in the CVOTs. Glycated hemoglobin (HbA_1c_), systolic blood pressure and pulse rate were broadly similar between the CVOTs and glycemic efficacy trials.

**Table 1:** Pooled baseline characteristics for the semaglutide trials

	CVOTs^a^		Glycemic efficacy trials^b^		Overall	
	Semaglutide	Placebo	Semaglutide	Comparator^c^	Semaglutide	Comparator^c^
	n = 3,239	n = 3,241	n = 7,269	n = 3,896	n = 10,508	n = 7,137
CV risk score	−1.0 (0.6)	−0.9 (0.6)	−1.7 (0.6)	−1.7 (0.6)	−1.5 (0.7)	−1.4 (0.7)
Age, years	65.3 (7.2)	65.5 (7.4)	57.5 (10.4)	57.5 (10.6)	59.9 (10.2)	61.1 (10.1)
HbA_1c_, %	8.4 (1.5)	8.4 (1.6)	8.2 (0.9)	8.2 (0.9)	8.3 (1.1)	8.3 (1.2)
Smoking status, n (%)						
Current smoker	388 (12.0)	367 (11.3)	1,254 (17.3)	667 (17.1)	1,642 (15.6)	1,034 (14.5)
Never smoked	1,473 (45.5)	1,457 (45.0)	3,946 (54.3)	2,153 (55.3)	5,419 (51.6)	3,610 (50.6)
Previous smoker	1,378 (42.5)	1,417 (43.7)	2,069 (28.5)	1,076 (27.6)	3,447 (32.8)	2,493 (34.9)
LDL-C, mmol/L	2.2 (0.9)	2.3 (0.9)	2.7 (0.9)	2.7 (0.9)	2.6 (0.9)	2.5 (0.9)
Pulse rate, bpm	71.6 (11.1)	71.5 (11.1)	74.2 (10.5)	74.3 (10.5)	73.4 (10.7)	73.0 (10.9)
Systolic BP, mmHg	135.7 (17.5)	135.5 (17.2)	132.2 (14.7)	132.3 (15.1)	133.3 (15.7)	133.8 (16.2)
Heart failure^d^, n (%)	564 (17.4)	586 (18.1)	349 (4.8)	212 (5.4)	913 (8.7)	798 (11.2)
NYHA						
Class I^d^	91 (2.8)	97 (3.0)	155 (2.1)	93 (2.4)	246 (2.3)	190 (2.7)
Class II	404 (12.5)	419 (12.9)	177 (2.4)	115 (3.0)	581 (5.5)	534 (7.5)
Class III	69 (2.1)	70 (2.2)	17 (0.2)	4 (0.1)	86 (0.8)	74 (1.0)
Prior ischemic heart disease, n (%)	1,403 (43.3)	1,430 (44.1)	850 (11.7)	491 (12.6)	2,253 (21.4)	1,921 (26.9)
Prior MI, n (%)	1,091 (33.7)	1,131 (34.9)	307 (4.2)	185 (4.7)	1,398 (13.3)	1,316 (18.4)
Prior stroke, n (%)	363 (11.2)	412 (12.7)	199 (2.7)	117 (3.0)	562 (5.3)	529 (7.4)
Insulin use, n (%)	1,740 (53.7)	1,722 (53.1)	869 (12.0)	374 (9.6)	2,609 (24.8)	2,096 (29.4)
eGFR,^e^ ml/min/1.73 m^2^	75.0 (21.8)	75.1 (22.1)	94.8 (17.1)	94.3 (17.9)	88.7 (20.8)	85.6 (22.1)

Data are mean (SD) unless otherwise stated

^a^CVOTs included SUSTAIN 6 and PIONEER 6.

^b^Glycemic efficacy trials included SUSTAIN 1–5, the SUSTAIN Japanese trials, PIONEER 1–5 and PIONEER 7–10.

^c^Comparators included placebo, sitagliptin, exenatide ER, insulin glargine, dulaglutide, liraglutide and empagliflozin; for details of the n, (%) and observation times for each comparator see Additional file 3: Appendix Table S3.

^d^The PIONEER 6 trial did not capture data on subjects with NYHA Class I.

^e^eGFR was estimated using the Chronic Kidney Disease Epidemiology Collaboration formula.

*BP* blood pressure, *bpm* beats per minute, *CV* cardiovascular, *CVOT* cardiovascular outcomes trial, *eGFR* estimated glomerular filtration rate, *exenatide ER* exenatide extended release, *HbA*_*1c*_ glycated hemoglobin, *LDL-C* low-density lipoprotein cholesterol, *MI* myocardial infarction, *NHYA* New York Heart Association, *SD* standard deviation

**Fig. 1:**
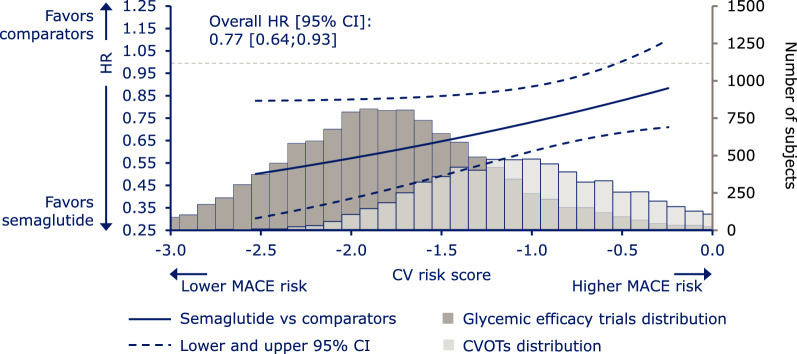
Relative risk of MACE as a function of baseline CV risk and distribution of subjects. Hazard ratio for treatment effect (semaglutide vs comparator) and 95% CI estimated using a stratified Cox proportional hazards model including effects of treatment, CV risk score and interaction between both. The x-axis shows the CV risk score derived from subjects’ baseline characteristics in the semaglutide trials. Data on graph cut off at the 5th and 95th percentile of the whole dataset. Hazard ratio value of 1.00 is indicated by a horizontal dashed line. Underlying histograms: distribution of subjects in the glycemic efficacy trials or CVOTs across baseline CV risk scores (histogram data for 439 subjects not shown, as these subjects had a CV risk score of < –3.0 or > 0.0). *CI* confidence interval, *CV* cardiovascular, *CVOT* cardiovascular outcomes trial, *HR* hazard ratio, *MACE* major adverse cardiovascular events

As expected, the average CV risk score was lower in the pooled glycemic efficacy trials than in the pooled CVOTs, but there was substantial overlap in the distributions (Fig. 1).

Subject numbers, observation times and number of MACE by individual trial are summarized in Additional file 3: Appendix Table S3. In individual trials, observation times were highest in the CVOTs (a total of 6,716 patient-years in SUSTAIN 6 and 4,182 patient-years in PIONEER 6); they were also high in the oral semaglutide arms of PIONEER 3 (2,148 patient-years), as this trial included three semaglutide treatment arms, each receiving a different dose of semaglutide. Across trials, observation times were higher with semaglutide (13,099 patient-years) than with comparator (9,434 patient-years); when examining individual comparators, observation times were highest for placebo, dipeptidyl peptidase-4 inhibitors and other GLP-1 receptor agonists (6,208, 1,527 and 883 patient-years, respectively).

Baseline subject characteristics by individual trial are summarized in Additional file 4: Appendix Table S4. At baseline, mean age and the proportion of subjects with heart failure and prior CV events were higher, and mean eGFR was lower, in the SUSTAIN 6, PIONEER 5 and PIONEER 6 trials than in the other trials. The proportions of subjects with heart failure (all classes) and Class II heart failure were also higher in these three trials than the other trials, while the proportions of subjects with Class III heart failure were higher in SUSTAIN 6 and PIONEER 6 than the other trials. These differences are not unexpected given that SUSTAIN 6 and PIONEER 6 included subjects at high risk of CVD, and PIONEER 5 included subjects with renal impairment. The proportion of insulin users was higher in the SUSTAIN 5 and 6 and PIONEER 5, 6 and 8 trials than in all other trials. LDL-cholesterol was generally higher in the Japanese trials than in the international trials. Smoking status varied between trials, and pulse rate was broadly similar across all trials. When assessing baseline characteristics by comparator (Additional file 4: Appendix Table S4C), the proportion of subjects with heart failure, other prior CV events and prior insulin use was higher, and mean eGFR was lower, in the placebo and semaglutide groups than in the other comparator groups, driven by the large patient numbers and high baseline CV risk in the CVOTs. Other baseline characteristics were broadly consistent across the different comparators.

### Application of the CV risk prediction model to the semaglutide data

The CV risk prediction model equations and CV risk scores (produced when the CV risk prediction model, developed using data from the LEADER CVOT, was applied to data from the semaglutide trials) are shown in Additional file 2: Appendix Table S2.

The LEADER-derived CV risk prediction model predicted CV risk in the semaglutide data satisfactorily (the AUC [95% CI] of 0.77 [0.74;0.79] indicated a fair predictive performance [32]; Fig. 1). When considered separately, AUC values for the CVOTs and glycemic efficacy trials were 0.68 [0.65;0.71] and 0.74 [0.69;0.79], respectively.

### Semaglutide effects on MACE by baseline CV risk scores

Observation times and patient numbers for the analysis of the effect of semaglutide vs comparators on the composite MACE endpoint are presented in Table 2. In the two CVOTs, total observation time and patient numbers were approximately equal between semaglutide and placebo; in the glycemic efficacy trials, total observation times and patient numbers were higher with semaglutide vs comparators, as some trials included two semaglutide groups but only one comparator group. Observation times and patient numbers for the analysis of the effect of semaglutide vs comparators on the individual components of MACE are presented in Additional file 5: Appendix Table S5, and show similar results to the composite MACE endpoint.

**Table 2:** Number of subjects experiencing first MACE and mean observation time in the semaglutide trials

	Semaglutide				Comparator			
	n	N	(%)	Observation time, total (mean), patient-years	n	N	(%)	Observation time, total (mean), patient-years
CVOTs	167	3,239	5.2	5,464 (1.69)	217	3,241	6.7	5,434 (1.68)
Glycemic efficacy trials	55	7,269	0.8	7,636 (1.05)	34	3,896	0.9	4,000 (1.03)
Overall	222	10,508	2.1	13,099 (1.25)	251	7,137	3.5	9,434 (1.32)

Observation time is curtailed at a maximum of 109 weeks to align with the analysis timeframe

% proportion of subjects, *CVOT* cardiovascular outcomes trial, *MACE* major adverse cardiovascular events, *n* number of subjects with events, *N* number of subjects in full analysis set

#### Relative MACE risk estimates for semaglutide vs comparators

There was a reduced relative risk of MACE with semaglutide vs comparators across the baseline CV risk continuum (Fig. 1), with a non-significant interaction p-value between CV risk score and treatment (p = 0.06), and a trend towards the largest relative CV benefits (i.e. lower hazard ratios) in those with the lowest CV risk score (i.e. lowest baseline CV risk).

**Fig. 2:**
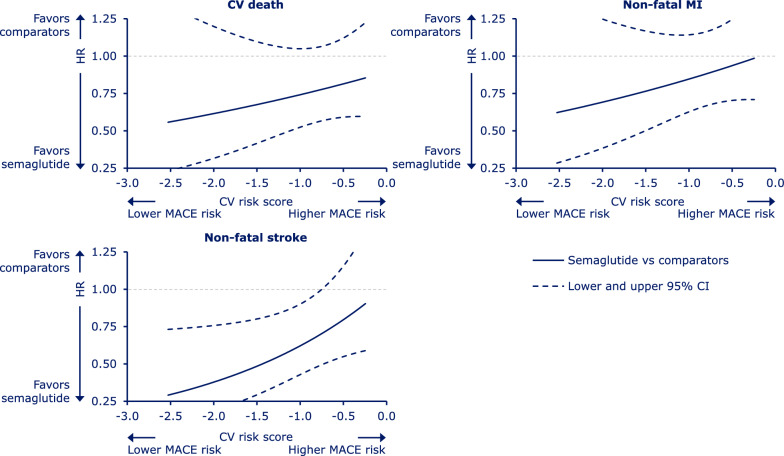
Relative risk of each individual MACE component as a function of CV risk. Hazard ratios for treatment effect (semaglutide vs comparators) across all SUSTAIN and PIONEER trials analyzed. Hazard ratios (semaglutide vs comparators) and 95% CIs estimated using a stratified Cox proportional hazards model including effects of treatment, CV risk score and interaction between both. The x-axis shows the CV risk score derived from subjects’ baseline characteristics in the semaglutide trials. Data on graph cut off at the 5th and 95th percentile of the whole dataset. Hazard ratio value of 1.00 is indicated by a horizontal dashed line. *CI* confidence interval, *CV* cardiovascular, *HR* hazard ratio, *MACE* major adverse cardiovascular events, *MI* myocardial infarction

The results for the individual MACE components are shown in Fig. 2. The shapes of the individual MACE component hazard ratio curves were similar to that of the 3-point composite MACE endpoint, which indicates consistent findings across the components.

In addition, when assessing the hazard ratios for MACE (and its components) for semaglutide vs comparators across the quartiles of CV risk, no significant interactions between treatment effect and baseline CV risk quartiles were found (Additional file 6: Appendix Figure S1). Typical subject profiles for each quartile of MACE risk are reported in Additional file 7: Appendix Table S6).

#### Absolute MACE risk estimates for semaglutide vs comparators

**Fig. 3:**
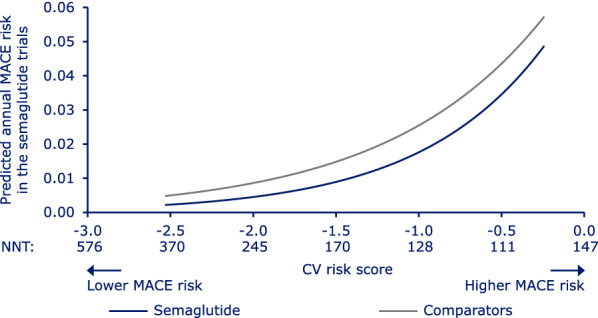
Estimated yearly risk of MACE as a function of CV risk. Absolute yearly MACE probabilities for semaglutide and comparators, respectively, estimated using a non-stratified Cox proportional hazards model including effects of treatment, CV risk score and interaction between both. The x-axis shows the CV risk score derived from subjects’ baseline characteristics in the semaglutide trials. Data on graph cut off at the 5th and 95th percentile of the whole dataset. *CV* cardiovascular, *MACE* major adverse cardiovascular events, *NNT* number needed to treat to avoid one MACE during 1 year

In concordance with the relative risk analysis, the absolute risk estimates for MACE with semaglutide vs comparators varied across the CV risk spectrum, with a trend for the largest absolute risk reduction in subjects at medium-to-high CV risk, as evidenced by the lowest NNT (111) being observed at a medium-to-high CV risk score of −0.483 (Fig. 3).

Similar patterns for treatment differences were observed when the absolute risk analysis was assessed by trial type (CVOTs or glycemic efficacy trials) or formulation (s.c. or oral), although there was a higher annual MACE risk in the CVOTs compared with the glycemic efficacy trials. In both CVOTs and glycemic efficacy trials, and with both s.c. and oral semaglutide, the greatest absolute MACE risk difference was in subjects with medium-to-high CV risk (Additional file 8: Appendix Figure S2).

#### Relative and absolute MACE risk estimates for semaglutide vs placebo

To remove the potential impact of the CV effects of active comparators on the analysis, a sensitivity analysis was conducted for semaglutide vs placebo comparator data, thereby excluding active comparator data (Additional file 9: Appendix Figure S3). This analysis yielded similar results to those already presented, indicating that the inclusion of comparators other than placebo had a limited effect on the results.

#### Subject profiles

The relevant baseline variables of sample subjects with low (5th percentile), midpoint and high (95th percentile) CV risk scores (two subjects each) are shown in Table 3, to illustrate example profiles associated with each CV risk score level. Hazard ratios (semaglutide vs comparators) for subjects included in the analysis ranged from 0.45 for the low CV risk profiles to 0.84 for the high CV risk profiles.

**Table 3:** Subject profiles, representing 5th percentile (low), midpoint and 95th percentile (high) CV risk

	Low CV risk		Midpoint CV risk		High CV risk	
	CV risk score: –2.53		CV risk score: –1.39		CV risk score: –0.25	
	NNT: 380		NNT: 159		NNT: 117	
Subject profile	A	B	C	D	E	F
Hazard ratio (semaglutide vs comparators)	0.45	0.45	0.62	0.62	0.84	0.84
Age, years	48	46	66	67	64	71
HbA_1c_, %	8.6	7.0	7.2	10.3	9.6	9.4
Smoking status, current/previous/never	Never	Never	Previous	Never	Previous	Never
LDL-C, mmol/L	3.3	3.3	3.2	3.9	4.5	5.2
Pulse rate, bpm	76	80	76	67	66	88
Systolic BP, mmHg	119	138	137	163	133	180
Prior ischemic heart disease, yes/no	No	No	No	No	No	Yes
Prior MI, yes/no	No	No	No	No	No	No
Prior stroke, yes/no	No	No	No	No	Yes	No
HF, NYHA class	No HF	No HF	No HF	No HF	Other^a^	II
Insulin use, yes/no	No	No	No	No	Yes	No
eGFR, ml/min/1.73 m^2^	117.7	116.2	91.7	94.1	88.9	66.9

Examples of real subject profiles were chosen at the 5th, midpoint and 95th percentiles of CV risk score distribution. The factors listed are those that were identified, based on data from LEADER, as having a significant effect on CV risk (no other factors were identified as important)

^a^Other: subject either had no HF or Class I HF

*BP* blood pressure, *bpm* beats per minute, *CV* cardiovascular, *eGFR* estimated glomerular filtration rate, *HbA*_*1c*_ glycated hemoglobin, *HF* heart failure, *LDL-C* low-density lipoprotein cholesterol, *MI* myocardial infarction, *NNT* numbers needed to treat, *NYHA* New York Heart Association

## Discussion

### Key findings

To allow a more granular analysis of the effect of semaglutide on MACE and its relation to baseline CV risk, a continuous CV risk prediction model was developed using data from a liraglutide CVOT (LEADER). This CV risk prediction model was used to distribute subjects by baseline CV risk in a pooled dataset including data for both s.c. and oral semaglutide. The CV risk prediction model predicted the risk of CV outcomes observed in the semaglutide trials fairly well, validating the selection of the LEADER model for this analysis.

The results showed that there was a reduced relative and absolute risk of MACE with semaglutide vs comparators across the spectrum of baseline CV risk scores. While relative risk reductions are typically independent of baseline risk in the population and absolute risk reductions are dependent on this, our data may also indicate some effect of baseline CV risk on relative risk reduction. Similar findings were also obtained for the individual MACE components when data were analyzed with only placebo as a comparator and when the results were analyzed by trial type. The results of the placebo sensitivity analysis were to be expected, because the majority of subjects receiving comparators (59%) were randomized to placebo rather than active comparators in the included trials.

Although the absolute risk reduction was small, there was a trend (p = 0.06) towards the largest relative risk reduction occurring in those with lowest CV risk. The explanatory mechanism underlying this observation is unclear. It could be hypothesized that more advanced disease may be more resistant (or very high-risk groups more non-responsive) to the beneficial effects of GLP-1 receptor agonists on CV outcomes.

### Findings in context of the broader literature

Our results are consistent overall with a post hoc analysis of pooled SUSTAIN and PIONEER data, which also showed that the effect of semaglutide vs comparators on MACE was largely consistent across different CV subgroups [35]. Furthermore, a meta-analysis including CVOTs for all GLP-1 receptor agonists found no significant heterogeneity in the effect of these therapies in subgroups with a history of CVD vs those with no history of CVD [36]. Although there were no significant subgroup interactions in the meta-analysis, there was a numerically lower risk of MACE in subjects with a history of CVD (−14%, hazard ratio 0.86) vs those without such a history (−6%, hazard ratio 0.94) [36]. The HARMONY Outcomes CVOT (which only included subjects with established CVD) similarly showed a 22% CV risk reduction with albiglutide vs placebo (hazard ratio 0.78) [37], and the REWIND CVOT showed an identical 13% effect size across subgroups with and without established CVD (hazard ratio 0.87) [38]. Taken together, these findings are counter to the suggestion that there is a greater relative CV benefit of GLP-1 receptor agonists in subjects at lower CV risk. Therefore, we believe the totality of data for GLP-1 receptor agonists indicates similar relative risk reduction effects across a broad continuum of baseline CV risk.

While the analysis presented here was not designed to assess the mechanisms by which semaglutide reduces CV risk, previous studies have revealed several potential beneficial effects. In an animal model of acute inflammation, semaglutide decreased levels of plasma markers of systemic inflammation and down-regulated multiple inflammatory pathways vs controls, and was associated with significant attenuation of plaque lesion development [39]. In clinical trials, semaglutide has provided clinically relevant reductions in CV risk factors such as excess body weight [8–30]. Weight loss with s.c. semaglutide was shown to be of a similar or greater magnitude compared with liraglutide 3.0 mg [40], and possibly more rapid – potentially as a result of the higher albumin affinity of semaglutide compared with liraglutide [41]. Furthermore, in subjects receiving liraglutide for an average of 4 years, switching to s.c. semaglutide has been shown to provide further reductions in HbA_1c_ [42]. Clinical trials have also demonstrated a beneficial effect of semaglutide on CV outcomes. In SUSTAIN 6, in addition to significantly reducing the risk of the primary outcome (3-point MACE: CV death, non-fatal stroke, non-fatal myocardial infarction), s.c. semaglutide significantly decreased the incidence of new or worsening nephropathy and of non-fatal stroke vs placebo [13]—the latter being a finding that has not been observed in CVOTs with other GLP-1 receptor agonists [43]. Nevertheless, it should be noted that a higher risk of diabetic retinopathy complications was observed with s.c. semaglutide vs placebo in SUSTAIN 6—a finding possibly related to the rapid initial reduction in HbA_1c_ with this GLP-1 receptor agonist [44]. A post hoc analysis of SUSTAIN 6 showed that the beneficial effect of s.c semaglutide vs placebo on MACE was not dependent on the gender, age or baseline CV risk profile of subjects [45].

Our findings with semaglutide are based on a CV risk prediction model derived from data from the LEADER CVOT; LEADER had common adjudicated endpoints, shared baseline variables and similar inclusion criteria, and evaluated a similar T2D population, to those included in the semaglutide trials. In line with this, when the CV risk prediction model was applied to the semaglutide dataset it performed satisfactorily, both overall and when applied only to data from the glycemic efficacy trials, validating the utility of the LEADER model for this analysis.

There are other risk models in the literature that could have been candidates for this analysis. However, each has limitations that made them less suited for our analyses compared with the CV risk prediction model developed using the LEADER data. The Framingham [46, 47] and SCORE [48] models were not used because they did not include patients with established CVD, which constituted the majority of subjects in the CVOTs in our analysis, as prior CVD is generally considered to be an important risk factor for new events [49]. Furthermore, an evaluation of the risk equations of models for CV risk (including Framingham and SCORE) has reported that these models do not provide reliable estimates of CV risk in patients with T2D [50]. The DIAL model was considered for use in our analysis, as it was developed with data from patients both with and without established CVD (19% and 81%, respectively) [51]. However, this was a community-based study and the translatability to a clinical trial population with adjudicated endpoints would be uncertain [51]. In addition, DIAL had a considerably longer follow-up time than LEADER (only 5- and 10-year predictions could be validated in the DIAL model) and some of the risk factors in the DIAL model (e.g. micro- and macroalbuminuria) were not available in the full semaglutide dataset.

### Strengths and limitations

A limitation of our study is that some baseline factors that may have been important predictors in the CV risk prediction model (e.g. urinary albumin-to-creatinine ratio) were not measured across all trials and, therefore, were not included in the analysis. Potential weaknesses of the LEADER model include that all patients were of high or higher CV risk than those in the SUSTAIN and PIONEER efficacy trials, which may have affected the analysis at the lower end of the continuum of risk. However, the AUC for the glycemic efficacy trials only was 0.74 [0.69;0.79], which is broadly in line with the AUC for the combined data set.

## Conclusion

This analysis suggests that semaglutide reduces the risk of MACE vs comparators across the continuum of baseline CV risk characterizing a broad T2D population. The results of this analysis will help enable physicians to understand the CV benefits of the GLP-1 analog semaglutide in patients with T2D across a broad continuum of CV risk.

## Supplementary information

**Additional file 1: Table S1.** Summary of LEADER model, including full details of the baseline variables considered in the model. Data are based on the full analysis set. Individual HRs for stroke, smoking status, eGFR and LDL-C are not presented, as these were influenced by other baseline parameters. bpm, beats per minute; CI, confidence interval; CKD-EPI, Chronic Kidney Disease Epidemiology Collaboration; eGFR, estimated glomerular filtration rate; HbA_1c_, glycated hemoglobin; HR, hazard ratio; LDL-C, low-density lipoprotein cholesterol; MI, myocardial infarction; NYHA, New York Heart Association; SBP, systolic blood pressure; SE, standard error.

**Additional file 2: Table S2.** Summary of risk models for semaglutide (including model selection) for pooled SUSTAIN and PIONEER data. AIC, Akaike Information Criterion; SBC, Schwarz Bayesian Information Criterion.

**Additional file 3: Table S3.** First MACE by individual trial in the semaglutide and comparator groups (A) and by drug class (B). *GLP-1RA comparator data; ^†^placebo comparator data. Observation time is curtailed at a maximum of 109 weeks to align with the analysis timeframe. %, proportion of subjects; DPP-4i, dipeptidyl peptidase-4 inhibitor; GLP-1RA, glucagon-like peptide-1 receptor agonist; JP, Japanese trial; MACE, major adverse cardiovascular events; Mono, monotherapy; n, number of subjects with events; N, number of subjects in full analysis set; OAD, oral antidiabetes drug; SGLT-2i, sodium–glucose co-transporter-2 inhibitor.

**Additional file 4: Table S4.** Baseline characteristics, observation times and number of events in subjects receiving semaglutide in individual SUSTAIN (A) and PIONEER (B) trials, and in subjects receiving comparators (C). A: *The CV risk score was derived as the predicted values from a Cox proportional hazard regression of time to first MACE, where all significant baseline predictors, except randomized treatment, were included as explanatory variables. ^†^eGFR was estimated using the CKD-EPI formula. Data are mean (SD) or n (%). bpm, beats per minute; CKD-EPI, Chronic Kidney Disease Epidemiology Collaboration; CV, cardiovascular; eGFR, estimated glomerular filtration rate; HbA_1c_, glycated hemoglobin; JP, Japanese trial; LDL-C, low-density lipoprotein cholesterol; MACE, major adverse cardiovascular events; MI, myocardial infarction; Mono, monotherapy; NHYA, New York Heart Association; OAD, oral antidiabetes drug; SBP, systolic blood pressure; SD, standard deviation. B: *The CV risk score was derived as the predicted values from a Cox proportional hazard regression of time to first MACE, where all significant baseline predictors, except randomized treatment, were included as explanatory variables. ^†^eGFR was estimated using the CKD-EPI formula. Data are mean (SD) or n (%). bpm, beats per minute; CKD-EPI, Chronic Kidney Disease Epidemiology Collaboration; CV, cardiovascular; eGFR, estimated glomerular filtration rate; HbA_1c_, glycated hemoglobin; LDL-C, low-density lipoprotein cholesterol; MACE, major adverse cardiovascular events; MI, myocardial infarction; NHYA, New York Heart Association; SBP, systolic blood pressure; SD, standard deviation. C: *The CV risk score was derived as the predicted values from a Cox proportional hazard regression of time to first MACE, where all significant baseline predictors, except randomized treatment, were included as explanatory variables. ^†^eGFR was estimated using the CKD-EPI formula. Data are mean (SD) or n (%). bpm, beats per minute; CKD-EPI, Chronic Kidney Disease Epidemiology Collaboration; CV, cardiovascular; DPP-4i, dipeptidyl peptidase-4 inhibitor; eGFR, estimated glomerular filtration rate; GLP-1RA, glucagon-like peptide-1 receptor agonist; HbA_1c_, glycated hemoglobin; HF, heart failure; LDL-C, low-density lipoprotein cholesterol; MACE, major adverse cardiovascular events; MI, myocardial infarction; NHYA, New York Heart Association; OAD, oral antidiabetes drug; SD, standard deviation; SGLT-2i, sodium–glucose co-transporter-2 inhibitor.

**Additional file 5: Table S5.** Time to first MACE by individual component in the semaglutide CVOTs and glycemic efficacy trials. Observation time is curtailed at a maximum of 109 weeks to align with the analysis timeframe. %, proportion of subjects; CV, cardiovascular; CVOT, cardiovascular outcomes trial; MACE, major adverse cardiovascular events; MI, myocardial infarction; n, number of subjects with events; N, number of subjects in full analysis set.

**Additional file 6: Figure S1.** Forest plots for semaglutide vs comparators across the quartiles of CV risk scores (and its components). *p-value for interaction between treatment and CV risk quartile. Data in parentheses are the risk scores included in each quartile. CI, confidence interval; CV cardiovascular; HR, hazard ratio; MACE, major adverse cardiovascular events; MI, myocardial infarction; Q, quartile.

**Additional file 7: Table S6.** Subject profiles, representing the four quartiles of baseline CV risk. Examples of real subject profiles were chosen at the 12.5th, 37.5th, 62.5th and 87.5th percentiles of CV risk score distribution. The factors listed are those that were identified, based on data from LEADER, as having a significant effect on CV risk (no other factors were identified as important). BP, blood pressure; bpm, beats per minute; CV, cardiovascular; eGFR, estimated glomerular filtration rate; HbA_1c_, glycated hemoglobin; LDL-C, low-density lipoprotein cholesterol; MACE, major adverse cardiovascular events; MI, myocardial infarction; NNT, number needed to treat to avoid one MACE during 1 year; NYHA, New York Heart Association.

**Additional file 8: Figure S2.** Absolute yearly risk of MACE with semaglutide vs comparators combined as a function of baseline CV risk for the CVOTs (A) and glycemic efficacy trials (B). Absolute yearly MACE probabilities, estimated using a stratified Cox proportional hazards model including effects of treatment, CV risk score and interaction between both. The x-axis shows the CV risk score derived from subjects’ baseline characteristics in the semaglutide trials. Data on graph cut off at the 5th and 95th percentile of whole dataset. CV, cardiovascular; CVOT, cardiovascular outcomes trial; MACE, major adverse cardiovascular events.

**Additional file 9: Figure S3.** Sensitivity analysis of relative (A) and absolute (B) MACE risk estimates for semaglutide vs placebo (excluding active comparators). Absolute yearly MACE probabilities, estimated using a stratified Cox proportional hazards model including effects of treatment, CV risk score and interaction between both. Includes placebo data from SUSTAIN 1, SUSTAIN 5, PIONEER 1, PIONEER 4, PIONEER 5, PIONEER 6, PIONEER 8 and PIONEER 9. Hazard ratio value of 1.00 is indicated by horizontal dashed line. For panel B the model was without the stratification. The x-axis shows the CV risk score derived from subjects’ baseline characteristics in the semaglutide trials. Data on graph cut off at the 5th and 95th percentile of whole dataset. CI, confidence interval; CV, cardiovascular; CVOT, cardiovascular outcomes trial; HR, hazard ratio; MACE, major adverse cardiovascular events; NNT, number needed to treat to avoid one MACE during 1 year.

## Data Availability

Data relating to this analysis will be made available upon request.

## Abbreviations

%: Proportion of subjects
AIC: Akaike information criterion
AUC: Area under the curve
BP: Blood pressure
bpm: Beats per minute
CKD-EPI: Chronic Kidney Disease Epidemiology Collaboration
CI: Confidence interval
CV: Cardiovascular
CVD: Cardiovascular disease
CVOT: Cardiovascular outcomes trial
DPP-4i: Dipeptidyl peptidase-4 inhibitor
eGFR: Estimated glomerular filtration rate
exenatide ER: Exenatide extended release
GLP-1: Glucagon-like peptide-1
GLP-1RA: Glucagon-like peptide-1 receptor agonist
HbA_1c_: Glycated hemoglobin
HF: Heart failure
HR: Hazard ratio
JP: Japanese trial
LDL-C: Low-density lipoprotein cholesterol
MACE: Major adverse cardiovascular events
MI: Myocardial infarction
Mono: Monotherapy
n: Number of subjects with events
N: Number of subjects in full analysis set
NNT: Number needed to treat to avoid one MACE during 1 year
NYHA: New York Heart Association
OAD: Oral antidiabetes drug
Q: Quartile
SBC: Schwarz Bayesian Information Criterion
SBP: Systolic blood pressure
SD: Standard deviation
SE: Standard error
SGLT-2i: Sodium-glucose co-transporter-2 inhibitor
T2D: Type 2 diabetes
