# Supplementary material for: Effects of semaglutide on risk of cardiovascular events across a continuum of cardiovascular risk: combined post hoc analysis of the SUSTAIN and PIONEER trials
Source: Cardiovasc Diabetol. 2020 Sep 30;19:156. doi: 10.1186/s12933-020-01106-4 (PMC7526237; doi:10.1186/s12933-020-01106-4)
Supplement: Supplementary file 1 — Additional file 1: Table S1. Summary of LEADER model, including full details of the baseline variables considered in the model. Data are based on the full analysis set. Individual HRs for stroke, smoking status, eGFR and LDL-C are not presented, as these were influenced by other baseline parameters. bpm, beats per minute; CI, confidence interval; CKD-EPI, Chronic Kidney Disease Epidemiology Collaboration; eGFR, estimated glomerular filtration rate; HbA1c, glycated hemoglobin; HR, hazard ratio; LDL-C, low-density lipoprotein cholesterol; MI, myocardial infarction; NYHA, New York Heart Association; SBP, systolic blood pressure; SE, standard error. [file 12933_2020_1106_MOESM1_ESM.docx]

**Supplementary Appendix Table S1.** Summary of LEADER model, including full details of the baseline variables considered in the model

|  | **Model term** | **Estimate (SE)** | **HR unit** | **HR**  **[95% CI]** | **p-value** |
| --- | --- | --- | --- | --- | --- |
| **Main and interaction effects** |  |  |  |  |  |
| Age at baseline, years | (AGE–61.744) | 0.0266 (0.0056) | 1 unit increase | 1.0270 [1.0158;1.0383] | <0.0001 |
| SBP at baseline, mmHg | (BPSYSBL–134.316) | 0.0069 (0.0020) | 1 unit increase | 1.0069 [1.0030;1.0109] | 0.0006 |
| HbA_1c_ at baseline, % | (HBA1CBL–8.417) | 0.0765 (0.0235) | 1 unit increase | 1.0795 [1.0310;1.1304] | 0.0011 |
| Use of insulin at baseline | (INSRANFL=No) | –0.1920 (0.0758) | No vs Yes | 0.8253 [0.7114;0.9574] | 0.0113 |
|  | (INSRANFL=Yes) | 0.0000 (–) |  |  |  |
| Prior ischemic heart disease | (ISCPRIFL=No) | –0.2184 (0.0835) | No vs Yes | 0.8038 [0.6826;0.9467] | 0.0089 |
|  | (ISCPRIFL=Yes) | 0.0000 (–) |  |  |  |
| Prior MI | (MIPRIFL=No) | –0.6287 (0.0778) | No vs Yes | 0.5333 [0.4579;0.6211] | <0.0001 |
|  | (MIPRIFL=Yes) | 0.0000 (–) |  |  |  |
| NYHA class | (NYHACLASS=Other incl NYHA class I) | 0.0000 (–) |  |  |  |
|  | (NYHACLASS=NYHA CLASS II) | 0.3016 (0.1049) | NYHA Class II vs Other (including NYHA class I) | 1.3521 [1.1008;1.6607] | 0.0040 |
|  | (NYHACLASS=NYHA CLASS III) | 0.2633 (0.2144) | NYHA Class III vs Other (including NYHA class I) | 1.3012 [0.8547;1.9810] | 0.2195 |
| Heart rate at baseline, bpm | (PULSEBL–73.009) | 0.0070 (0.0033) | 1 unit increase | 1.0071 [1.0007;1.0135] | 0.0305 |
| LDL-C at baseline x Smoking status as baseline | (LDLBL–2.456) x (SMOKER=CURRENT SMOKER) | 0.3441 (0.0959) | 1 unit increase when smoking status at baseline was ’Current Smoker’ | 1.4107 [1.1690;1.7023] | 0.0003 |
|  | (LDLBL–2.456) x (SMOKER=NEVER SMOKED) | 0.0204 (0.0663) | 1 unit increase when smoking status at baseline was ‘Never Smoked’ | 1.0206 [0.8963;1.1623] | 0.7580 |
|  | (LDLBL–2.456) x (SMOKER=PREVIOUS SMOKER) | 0.2447 (0.0524) | 1 unit increase when smoking status at baseline was ’Previous Smoker’ | 1.2772 [1.1525;1.4154] | <0.0001 |
| Smoking status at baseline | (SMOKER=CURRENT SMOKER) | 0.2795 (0.1128) |  |  |  |
|  | (SMOKER=NEVER SMOKED) | –0.2037 (0.0827) |  |  |  |
|  | (SMOKER=PREVIOUS SMOKER) | 0.0000 (–) |  |  |  |
| eGFR (CKD-EPI) at baseline x  Prior stroke | (EGFRBL–84.551) x (STRPRIFL=No) | –0.0107 (0.0019) | 1 unit increase with no prior stroke | 0.9894 [0.9856;0.9932] | <0.0001 |
|  | (EGFRBL–84.551) x (STRPRIFL=Yes) | 0.0005 (0.0036) | 1 unit increase with prior stroke | 1.0005 [0.9934;1.0076] | 0.8947 |
| Prior stroke | (STRPRIFL=No) | –0.5141 (0.0974) |  |  |  |
|  | (STRPRIFL=Yes) | 0.0000 (–) |  |  |  |

Data are based on the full analysis set. Individual HRs for stroke, smoking status, eGFR and LDL-C are not presented, as these were influenced by other baseline parameters. bpm, beats per minute; CI, confidence interval; CKD-EPI, Chronic Kidney Disease Epidemiology Collaboration; eGFR, estimated glomerular filtration rate; HbA_1c_, glycated hemoglobin; HR, hazard ratio; LDL-C, low-density lipoprotein cholesterol; MI, myocardial infarction;
NYHA, New York Heart Association; SBP, systolic blood pressure; SE, standard error.
