# Supplementary material for: Effects of semaglutide on risk of cardiovascular events across a continuum of cardiovascular risk: combined post hoc analysis of the SUSTAIN and PIONEER trials
Source: Cardiovasc Diabetol. 2020 Sep 30;19:156. doi: 10.1186/s12933-020-01106-4 (PMC7526237; doi:10.1186/s12933-020-01106-4)
Supplement: Supplementary file 2 — Additional file 2: Table S2. Summary of risk models for semaglutide (including model selection) for pooled SUSTAIN and PIONEER data. AIC, Akaike Information Criterion; SBC, Schwarz Bayesian Information Criterion. [file 12933_2020_1106_MOESM2_ESM.docx]

**Supplementary Appendix Table S2.** Summary of risk models for semaglutide (including model selection) for pooled SUSTAIN and PIONEER data

| **Model terms** | **Model estimates** | **AIC** | **SBC** |
| --- | --- | --- | --- |
| Treatment only | –0.4420 x (Treatment=Semaglutide) | 8788.604 | 8792.763 |
| Risk score only | 1.2431 x risk score | 8443.532 | 8447.691 |
| Treatment and risk score (linear) with no interaction | –0.2972 x (Treatment=Semaglutide)  + 1.2281 x risk score | 8435.167 | 8443.485 |
| Treatment and risk score (linear) with interaction | –0.1003 x (Treatment=Semaglutide)  + 1.0929 x risk score + 0.2724 x risk score x (Treatment=Semaglutide) | 8432.449 | 8444.926 |

AIC, Akaike Information Criterion; SBC, Schwarz Bayesian Information Criterion.
