# Supplementary material for: Effects of semaglutide on risk of cardiovascular events across a continuum of cardiovascular risk: combined post hoc analysis of the SUSTAIN and PIONEER trials
Source: Cardiovasc Diabetol. 2020 Sep 30;19:156. doi: 10.1186/s12933-020-01106-4 (PMC7526237; doi:10.1186/s12933-020-01106-4)
Supplement: Supplementary file 3 — Additional file 3: Table S3. First MACE by individual trial in the semaglutide and comparator groups (A) and by drug class (B). *GLP-1RA comparator data; †placebo comparator data. Observation time is curtailed at a maximum of 109 weeks to align with the analysis timeframe. %, proportion of subjects; DPP-4i, dipeptidyl peptidase-4 inhibitor; GLP-1RA, glucagon-like peptide-1 receptor agonist; JP, Japanese trial; MACE, major adverse cardiovascular events; Mono, monotherapy; n, number of subjects with events; N, number of subjects in full analysis set; OAD, oral antidiabetes drug; SGLT-2i, sodium–glucose co-transporter-2 inhibitor. [file 12933_2020_1106_MOESM3_ESM.docx]

**Supplementary Appendix Table S3.** First MACE by individual trial in the semaglutide and comparator groups (A) and by drug class (B)

A.

|  | **Semaglutide** | | | | **Comparator** | | | |
| --- | --- | --- | --- | --- | --- | --- | --- | --- |
|  | **n** | **N** | **(%)** | **Observation time, total (mean), patient-years** | **n** | **N** | **(%)** | **Observation time, total (mean),  patient-years** |
| SUSTAIN 1 | 1 | 258 | (0.4) | 172 (0.67) | 0 | 129 | (0.0) | 85 (0.66) |
| SUSTAIN 2 | 4 | 818 | (0.5) | 938 (1.15) | 4 | 407 | (1.0) | 468 (1.15) |
| SUSTAIN 3 | 1 | 404 | (0.2) | 460 (1.14) | 2 | 405 | (0.5) | 458 (1.13) |
| SUSTAIN 4 | 4 | 722 | (0.6) | 475 (0.66) | 2 | 360 | (0.6) | 239 (0.66) |
| SUSTAIN 5 | 2 | 263 | (0.8) | 175 (0.67) | 0 | 133 | (0.0) | 87 (0.65) |
| SUSTAIN 6 | 106 | 1,648 | (6.4) | 3,362 (2.04) | 141 | 1,649 | (8.6) | 3,353 (2.03) |
| SUSTAIN JP OAD | 0 | 480 | (0.0) | 555 (1.16) | 0 | 120 | (0.0) | 139 (1.16) |
| SUSTAIN JP Mono | 1 | 205 | (0.5) | 138 (0.67) | 0 | 103 | (0.0) | 70 (0.68) |
| PIONEER 1 | 2 | 525 | (0.4) | 309 (0.59) | 2 | 178 | (1.1) | 105 (0.59) |
| PIONEER 2 | 3 | 411 | (0.7) | 442 (1.08) | 5 | 410 | (1.2) | 439 (1.07) |
| PIONEER 3 | 16 | 1,396 | (1.1) | 2,148 (1.54) | 5 | 467 | (1.1) | 731 (1.57) |
| PIONEER 4 | 4 | 285 | (1.4) | 307 (1.08) | 4 | 426 | (0.9) | 304 (1.07)* 150 (1.06)^†^ |
| PIONEER 5 | 2 | 163 | (1.2) | 95 (0.58) | 2 | 161 | (1.2) | 96 (0.59) |
| PIONEER 6 | 61 | 1,591 | (3.8) | 2,101 (1.32) | 76 | 1,592 | (4.8) | 2,081 (1.31) |
| PIONEER 7 | 1 | 253 | (0.4) | 259 (1.02) | 3 | 251 | (1.2) | 258 (1.03) |
| PIONEER 8 | 11 | 547 | (2.0) | 583 (1.06) | 5 | 184 | (2.7) | 198 (1.08) |
| PIONEER 9 | 0 | 146 | (0.0) | 156 (1.07) | 0 | 97 | (0.0) | 52 (1.08)* 54 (1.09)^†^ |
| PIONEER 10 | 3 | 393 | (0.8) | 422 (1.08) | 0 | 65 | (0.0) | 68.9 (1.06) |

B.

|  | **n** | **N** | **Observation time, total (mean), patient-years** |
| --- | --- | --- | --- |
| Semaglutide | 222 | 10,508 | 13,099 (1.25) |
| Placebo | 228 | 4,217 | 6,208 (1.47) |
| DPP-4i | 12 | 1,228 | 1,527 (1.24) |
| GLP-1RA | 4 | 802 | 883 (1.10) |
| SGLT-2i | 5 | 410 | 439 (1.07) |
| Insulins | 2 | 360 | 239 (0.66) |
| OADs | 0 | 120 | 139 (1.16) |

*GLP-1RA comparator data; ^†^placebo comparator data. Observation time is curtailed at a maximum of 109 weeks to align with the analysis timeframe. %, proportion of subjects; DPP-4i, dipeptidyl peptidase-4 inhibitor; GLP-1RA, glucagon-like peptide-1 receptor agonist; JP, Japanese trial; MACE, major adverse cardiovascular event; Mono, monotherapy; n, number of subjects with events; N, number of subjects in full analysis set; OAD, oral antidiabetes drug; SGLT-2i, sodium–glucose co-transporter-2 inhibitor.
