# Supplementary material for: Effects of semaglutide on risk of cardiovascular events across a continuum of cardiovascular risk: combined post hoc analysis of the SUSTAIN and PIONEER trials
Source: Cardiovasc Diabetol. 2020 Sep 30;19:156. doi: 10.1186/s12933-020-01106-4 (PMC7526237; doi:10.1186/s12933-020-01106-4)
Supplement: Supplementary file 4 — Additional file 4: Table S4. Baseline characteristics, observation times and number of events in subjects receiving semaglutide in individual SUSTAIN (A) and PIONEER (B) trials, and in subjects receiving comparators (C). A: *The CV risk score was derived as the predicted values from a Cox proportional hazard regression of time to first MACE, where all significant baseline predictors, except randomized treatment, were included as explanatory variables. †eGFR was estimated using the CKD-EPI formula. Data are mean (SD) or n (%). bpm, beats per minute; CKD-EPI, Chronic Kidney Disease Epidemiology Collaboration; CV, cardiovascular; eGFR, estimated glomerular filtration rate; HbA1c, glycated hemoglobin; JP, Japanese trial; LDL-C, low-density lipoprotein cholesterol; MACE, major adverse cardiovascular events; MI, myocardial infarction; Mono, monotherapy; NHYA, New York Heart Association; OAD, oral antidiabetes drug; SBP, systolic blood pressure; SD, standard deviation. B: *The CV risk score was derived as the predicted values from a Cox proportional hazard regression of time to first MACE, where all significant baseline predictors, except randomized treatment, were included as explanatory variables. †eGFR was estimated using the CKD-EPI formula. Data are mean (SD) or n (%). bpm, beats per minute; CKD-EPI, Chronic Kidney Disease Epidemiology Collaboration; CV, cardiovascular; eGFR, estimated glomerular filtration rate; HbA1c, glycated hemoglobin; LDL-C, low-density lipoprotein cholesterol; MACE, major adverse cardiovascular events; MI, myocardial infarction; NHYA, New York Heart Association; SBP, systolic blood pressure; SD, standard deviation. C: *The CV risk score was derived as the predicted values from a Cox proportional hazard regression of time to first MACE, where all significant baseline predictors, except randomized treatment, were included as explanatory variables. †eGFR was estimated using the CKD-EPI formula. Data are mean (SD) or n (%). bpm, beats per minute; C [file 12933_2020_1106_MOESM4_ESM.docx]

**Supplementary Appendix Table S4.** Baseline characteristics, observation times and number of events in subjects receiving semaglutide in individual SUSTAIN (A) and PIONEER (B) trials, and in subjects receiving comparators (C)

A.

| CV risk score* | **SUSTAIN 1** n=258  –1.9 (0.6) | **SUSTAIN 2** n=818  –1.9 (0.5) | **SUSTAIN 3** n=404  –1.8 (0.5) | **SUSTAIN 4** n=722  –1.9 (0.5) | **SUSTAIN 5** n=263  –1.4 (0.5) | **SUSTAIN 6** n=1,648  –0.9 (0.6) | **SUSTAIN JP Mono**  n=205  –1.7 (0.5) | **SUSTAIN JP OAD**  n=480  –1.7 (0.5) |
| --- | --- | --- | --- | --- | --- | --- | --- | --- |
| Age (years) | 53.6 (11.5) | 55.3 (9.8) | 56.3 (10.3) | 56.6 (10.3) | 58.8 (9.7) | 64.6 (7.2) | 58.4 (11.0) | 58.3 (10.4) |
| HbA_1c_ (%) | 8.1 (0.9) | 8.0 (0.9) | 8.4 (0.9) | 8.2 (0.9) | 8.3 (0.8) | 8.7 (1.5) | 8.1 (0.9) | 8.1 (0.9) |
| Smoking status  (n [%])  Current smoker  Never smoked  Previous smoker | 56 (21.7) 145 (56.2) 57 (22.1) | 141 (17.2) 495 (60.5) 182 (22.2) | 60 (14.9) 208 (51.5) 136 (33.7) | 100 (13.9) 430 (59.6) 192 (26.6) | 53 (20.2) 125 (47.5) 85 (32.3) | 204 (12.4) 754 (45.8) 690 (41.9) | 49 (23.9) 81 (39.5) 75 (36.6) | 131 (27.3) 184 (38.3) 165 (34.4) |
| LDL-C (mmol/L) | 2.8 (0.9) | 2.7 (0.9) | 2.7 (1.0) | 2.5 (0.9) | 2.6 (0.9) | 2.3 (0.9) | 3.0 (0.7) | 2.8 (0.7) |
| Pulse rate (bpm) | 74.3 (10.6) | 75.8 (9.9) | 75.2 (10.5) | 74.2 (10.2) | 73.3 (10.6) | 72.1 (11.1) | 72.2 (10.8) | 72.8 (10.5) |
| SBP (mmHg) | 128.4 (13.0) | 132.6 (15.0) | 133.4 (14.9) | 131.9 (15.1) | 134.6 (15.6) | 136.0 (17.5) | 130.3 (15.0) | 129.3 (13.1) |
| Heart failure, n (%)  NYHA class  Class I  Class II  Class III | 9 (3.5)  3 (1.2) 5 (1.9) 1 (0.4) | 43 (5.3)  18 (2.2) 20 (2.4) 5 (0.6) | 5 (1.2)  3 (0.7) 2 (0.5) 0 (0.0) | 14 (1.9)  5 (0.7) 9 (1.2) 0 (0.0) | 13 (4.9)  5 (1.9) 7 (2.7) 1 (0.4) | 376 (22.8)  91 (5.5) 241 (14.6) 44 (2.7) | 0 (0.0)  0 (0.0) 0 (0.0) 0 (0.0) | 0 (0.0)  0 (0.0) 0 (0.0) 0 (0.0) |
| Prior ischemic heart disease, n (%) | 16 (6.2) | 103 (12.6) | 31 (7.7) | 58 (8.0) | 39 (14.8) | 988 (60.0) | 3 (1.5) | 15 (3.1) |
| Prior MI, n (%) | 6 (2.3) | 22 (2.7) | 10 (2.5) | 25 (3.5) | 14 (5.3) | 530 (32.2) | 1 (0.5) | 7 (1.5) |
| Prior stroke, n (%) | 5 (1.9) | 15 (1.8) | 4 (1.0) | 8 (1.1) | 6 (2.3) | 191 (11.6) | 5 (2.4) | 19 (4.0) |
| Insulin use, n (%) | 0 (0.0) | 0 (0.0) | 0 (0.0) | 0 (0.0) | 263 (100.0) | 772 (46.8) | 0 (0.0) | 0 (0.0) |
| eGFR^†^ (ml/min/1.73 m^2^) | 97.3 (18.6) | 98.0 (14.4) | 97.6 (15.4) | 95.6 (18.1) | 90.4 (18.3) | 75.8 (22.5) | 96.5 (13.3) | 97.0 (14.4) |

*The CV risk score was derived as the predicted values from a Cox proportional hazard regression of time to first MACE, where all significant baseline predictors, except randomized treatment, were included as explanatory variables. ^†^eGFR was estimated using the CKD-EPI formula. Data are mean (SD) or n (%). bpm, beats per minute; CKD-EPI, Chronic Kidney Disease Epidemiology Collaboration; CV, cardiovascular; eGFR, estimated glomerular filtration rate; HbA_1c_, glycated hemoglobin; JP, Japanese trial; LDL-C, low-density lipoprotein cholesterol; MACE, major adverse cardiovascular events;
MI, myocardial infarction; Mono, monotherapy; NHYA, New York Heart Association; OAD, oral antidiabetes drug; SBP, systolic blood pressure;
SD, standard deviation.

B.

| CV risk score* | **PIONEER 1**  n=525  –1.9 (0.6) | **PIONEER  2**  n=411  –1.8 (0.6) | **PIONEER 3**  n=1,396  –1.7 (0.6) | **PIONEER  4**  n=285  –1.8 (0.6) | **PIONEER  5**  n=163  –0.7 (0.5) | **PIONEER  6**  n=1,591  –1.0 (0.5) | **PIONEER  7**  n=253  –1.8 (0.6) | **PIONEER  8**  n=547  –1.4 (0.6) | **PIONEER  9**  n=146  –1.7 (0.5) | **PIONEER 10**  n=393  –1.6 (0.5) |
| --- | --- | --- | --- | --- | --- | --- | --- | --- | --- | --- |
| Age (years) | 54.8 (11.0) | 57.4 (9.9) | 57.8 (9.9) | 56.2 (9.5) | 70.9 (7.9) | 65.9 (7.2) | 56.9 (9.7) | 60.8 (9.7) | 59.6 (9.5) | 58.1 (10.5) |
| HbA_1c_ (%) | 8.0 (0.7) | 8.1 (0.9) | 8.3 (0.9) | 8.0 (0.7) | 8.0 (0.7) | 8.2 (1.6) | 8.3 (0.6) | 8.2 (0.7) | 8.2 (0.9) | 8.3 (0.9) |
| Smoking status  (n [%])  Current smoker  Never smoked  Previous smoker | 90 (17.1) 339 (64.6) 96 (18.3) | 61 (14.8) 235 (57.2) 115 (28.0) | 201 (14.4) 783 (56.1) 412 (29.5) | 43 (15.1) 162 (56.8) 80 (28.1) | 8 (4.9) 106 (65.0) 49 (30.1) | 184 (11.6) 719 (45.2) 688 (43.2) | 40 (15.8) 139 (54.9) 74 (29.2) | 77 (14.1) 308 (56.3) 162 (29.6) | 39 (26.7) 60 (41.1) 47 (32.2) | 105 (26.7) 146 (37.2) 142 (36.1) |
| LDL-C (mmol/L) | 3.0 (0.9) | 2.6 (0.9) | 2.5 (0.9) | 2.6 (0.9) | 2.4 (0.9) | 2.2 (0.9) | 2.6 (1.0) | 2.5 (0.9) | 3.3 (0.7) | 3.1 (0.7) |
| Pulse rate (bpm) | 73.6 (10.5) | 74.3 (9.9) | 74.3 (10.7) | 74.3 (10.3) | 69.2 (10.6) | 71.0 (11.1) | 73.9 (10.2) | 74.6 (10.3) | 72.1 (10.2) | 74.9 (11.6) |
| SBP (mmHg) | 130.1 (13.8) | 132.4 (14.8) | 133.9 (15.1) | 132.2 (13.0) | 138.6 (15.5) | 135.5 (17.6) | 131.6 (14.6) | 133.1 (14.2) | 127.6 (13.3) | 130.8 (14.0) |
| Heart failure, n (%)  NYHA class  Class I  Class II  Class III | 16 (3.0)  11 (2.1) 5 (1.0) 0 (0.0) | 22 (5.4)  9 (2.2) 12 (2.9) 1 (0.2) | 112 (8.0)  48 (3.4) 56 (4.0) 8 (0.6) | 19 (6.7)  7 (2.5) 12 (4.2) 0 (0.0) | 56 (34.4)  21 (12.9) 35 (21.5) 0 (0.0) | 188 (11.8)  0 (0.0) 163 (10.2) 25 (1.6) | 3 (1.2)  0 (0.0) 3 (1.2) 0 (0.0) | 34 (6.2)  22 (4.0) 11 (2.0) 1 (0.2) | 0 (0.0)  0 (0.0) 0 (0.0) 0 (0.0) | 3 (0.8)  3 (0.8) 0 (0.0) 0 (0.0) |
| Prior ischemic heart disease, n (%) | 45 (8.6) | 57 (13.9) | 226 (16.2) | 38 (13.3) | 75 (46.0) | 415 (26.1) | 29 (11.5) | 104 (19.0) | 0 (0.0) | 11 (2.8) |
| Prior MI, n (%) | 13 (2.5) | 29 (7.1) | 81 (5.8) | 11 (3.9) | 31 (19.0) | 561 (35.3) | 15 (5.9) | 37 (6.8) | 0 (0.0) | 5 (1.3) |
| Prior stroke, n (%) | 7 (1.3) | 10 (2.4) | 34 (2.4) | 12 (4.2) | 11 (6.7) | 172 (10.8) | 9 (3.6) | 33 (6.0) | 4 (2.7) | 17 (4.3) |
| Insulin use, n (%) | 0 (0.0) | 0 (0.0) | 0 (0.0) | 0 (0.0) | 59 (36.2) | 968 (60.8) | 0 (0.0) | 547 (100.0) | 0 (0.0) | 0 (0.0) |
| eGFR^†^ (ml/min/1.73 m^2^) | 96.8 (15.2) | 96.1 (15.2) | 95.6 (15.7) | 95.9 (14.7) | 46.8 (9.7) | 74.2 (21.1) | 96.9 (14.4) | 91.6 (15.3) | 96.3 (13.0) | 96.6 (13.5) |

*The CV risk score was derived as the predicted values from a Cox proportional hazard regression of time to first MACE, where all significant baseline predictors, except randomized treatment, were included as explanatory variables. ^†^eGFR was estimated using the CKD-EPI formula. Data are mean (SD) or n (%). bpm, beats per minute; CKD-EPI, Chronic Kidney Disease Epidemiology Collaboration; CV, cardiovascular; eGFR, estimated glomerular filtration rate; HbA_1c_, glycated hemoglobin; LDL-C, low-density lipoprotein cholesterol; MACE, major adverse cardiovascular events; MI, myocardial infarction; NHYA, New York Heart Association; SBP, systolic blood pressure; SD, standard deviation.

C.

| CV risk score* | **Semaglutide** n=10,508  –1.5 (0.7) | **Placebo** n=4,217  –1.1 (0.7) | **DPP-4i** n=1,228  –1.8 (0.6) | **GLP-1RAs** n=802  –1.8 (0.6) | **SGLT-2i** n=410  –1.8 (0.5) | **Insulins** n=360  –1.9 (0.6) | **OADs** n=120  –1.7 (0.5) |
| --- | --- | --- | --- | --- | --- | --- | --- |
| Age (years) | 59.9 (10.2) | 64.0 (8.9) | 56.9 (10.2) | 57.0 (10.4) | 57.8 (10.0) | 56.1 (10.6) | 59.2 (10.1) |
| HbA_1c_ (%) | 8.3 (1.1) | 8.3 (1.4) | 8.2 (0.8) | 8.2 (0.9) | 8.1 (0.9) | 8.1 (0.9) | 8.1 (0.9) |
| Smoking status  (n [%])  Current smoker  Never smoked  Previous smoker | 1,642 (15.6) 5,419 (51.6) 3,447 (32.8) | 528 (12.5) 2,035 (48.3) 1,654 (39.2) | 202 (16.4) 694 (56.5) 332 (27.0) | 157 (19.6) 390 (48.6) 255 (31.8) | 59 (14.4) 228 (55.6) 123 (30.0) | 60 (16.7) 222 (61.7) 78 (21.7) | 28 (23.3)  41 (34.2)  51 (42.5) |
| LDL-C (mmol/L) | 2.6 (0.9) | 2.4 (0.9) | 2.6 (0.9) | 2.7 (0.9) | 2.7 (0.9) | 2.5 (0.9) | 2.9 0.7) |
| Pulse rate (bpm) | 73.4 (10.7) | 71.8 (11.0) | 75.1 (10.6) | 75.0 (10.6) | 73.9 (9.7) | 75.1 (10.2) | 72.7 (10.5) |
| SBP (mmHg) | 133.3 (15.7) | 134.7 (16.8) | 132.6 (15.7) | 132.7 (14.1) | 131.9 (14.6) | 132.4 (15.8) | 129.0 (12.6) |
| Heart failure, n (%)  NYHA class  Class I   Class II  Class III | 913 (8.7)  246 (2.3) 581 (5.5) 86 (0.8) | 674 (16.0)  132 (3.1) 470 (11.1) 72 (1.7) | 76 (6.2)  38 (3.1) 37 (3.0)  1 (<0.1) | 26 (3.2)  13 (1.6) 12 (1.5)  1 (0.1) | 16 (3.9)  4 (1.0)  12 (2.9) 0 (0.0) | 6 (1.7)  3 (0.8)  3 (0.8)  0 (0.0) | 0 (0.0)  0 (0.0)  0 (0.0)  0 (0.0) |
| Prior ischemic heart disease, n (%) | 2,253 (21.4) | 1,593 (37.8) | 168 (13.7) | 77 (9.6) | 46 (11.2) | 34 (9.4) | 3 (2.5) |
| Prior MI, n (%) | 1,398 (13.3) | 1,196 (28.4) | 52 (4.2) | 27 (3.4) | 22 (5.4) | 18 (5.0) | 1 (0.8) |
| Prior stroke, n (%) | 562 (5.3) | 448 (10.6) | 36 (2.9) | 23 (2.9) | 8 (2.0) | 9 (2.5) | 5 (4.2) |
| Insulin use, n (%) | 2,609 (24.8) | 2,094 (49.7) | 1 (<0.1) | 1 (0.1) | 0 (0.0) | 0 (0.0) | 0 (0.0) |
| eGFR^†^ (ml/min/1.73 m^2^) | 88.7 (20.8) | 77.9 (22.9) | 97.0 (14.7) | 96.8 (14.4) | 94.6 (15.0) | 97.1 (17.0) | 96.9 (14.1) |

*The CV risk score was derived as the predicted values from a Cox proportional hazard regression of time to first MACE, where all significant baseline predictors, except randomized treatment, were included as explanatory variables. ^†^eGFR was estimated using the CKD-EPI formula. Data are mean (SD) or n (%). bpm, beats per minute; CKD-EPI, Chronic Kidney Disease Epidemiology Collaboration; CV, cardiovascular; DPP-4i, dipeptidyl peptidase-4 inhibitor; eGFR, estimated glomerular filtration rate; GLP-1RA, glucagon-like peptide-1 receptor agonist; HbA_1c_, glycated hemoglobin; HF, heart failure; LDL-C, low-density lipoprotein cholesterol; MACE, major adverse cardiovascular events; MI, myocardial infarction; NHYA, New York Heart Association; OAD, oral antidiabetes drug; SD, standard deviation; SGLT-2i, sodium–glucose co-transporter-2 inhibitor.
