# Supplementary material for: Effects of semaglutide on risk of cardiovascular events across a continuum of cardiovascular risk: combined post hoc analysis of the SUSTAIN and PIONEER trials
Source: Cardiovasc Diabetol. 2020 Sep 30;19:156. doi: 10.1186/s12933-020-01106-4 (PMC7526237; doi:10.1186/s12933-020-01106-4)
Supplement: Supplementary file 5 — Additional file 5: Table S5. Time to first MACE by individual component in the semaglutide CVOTs and glycemic efficacy trials. Observation time is curtailed at a maximum of 109 weeks to align with the analysis timeframe. %, proportion of subjects; CV, cardiovascular; CVOT, cardiovascular outcomes trial; MACE, major adverse cardiovascular events; MI, myocardial infarction; n, number of subjects with events; N, number of subjects in full analysis set. [file 12933_2020_1106_MOESM5_ESM.docx]

**Supplementary Appendix Table S5.** Time to first MACE by individual component in the semaglutide CVOTs and glycemic efficacy trials

|  | **Semaglutide** | | | | **Comparator** | | | |
| --- | --- | --- | --- | --- | --- | --- | --- | --- |
|  | **n** | **N** | **(%)** | **Observation time, total (mean), patient-years** | **n** | **N** | **(%)** | **Observation time, total (mean),  patient-years** |
| **CV death** |  |  |  |  |  |  |  |  |
| CVOTs | 58 | 3,239 | (1.8) | 5,464 (1.69) | 73 | 3,241 | (2.3) | 5,434 (1.68) |
| Glycemic efficacy trials | 17 | 7,269 | (0.2) | 7,636 (1.05) | 12 | 3,896 | (0.3) | 4,000 (1.03) |
| Overall | 75 | 10,508 | (0.7) | 13,099 (1.25) | 85 | 7,137 | (1.2) | 9,434 (1.32) |
| **Non-fatal MI** |  |  |  |  |  |  |  |  |
| CVOTs | 82 | 3,239 | (2.5) | 5,464 (1.69) | 94 | 3,241 | (2.9) | 5,434 (1.68) |
| Glycemic efficacy trials | 20 | 7,269 | (0.3) | 7,636 (1.05) | 10 | 3,896 | (0.3) | 4,000 (1.03) |
| Overall | 102 | 10,508 | (1.0) | 13,099 (1.25) | 104 | 7,137 | (1.5) | 9,434 (1.32) |
| **Non-fatal stroke** |  |  |  |  |  |  |  |  |
| CVOTs | 39 | 3,239 | (1.2) | 5,464 (1.69) | 59 | 3,241 | (1.8) | 5,434 (1.68) |
| Glycemic efficacy trials | 19 | 7,269 | (0.3) | 7,636 (1.05) | 12 | 3,896 | (0.3) | 4,000 (1.03) |
| Overall | 58 | 10,508 | (0.6) | 13,099 (1.25) | 71 | 7,137 | (1.0) | 9,434 (1.32) |

Observation time is curtailed at a maximum of 109 weeks to align with the analysis timeframe. %, proportion of subjects; CV, cardiovascular;
CVOT, cardiovascular outcomes trial; MACE, major adverse cardiovascular events; MI, myocardial infarction; n, number of subjects with events;
N, number of subjects in full analysis set.
