# Supplementary material for: Effects of semaglutide on risk of cardiovascular events across a continuum of cardiovascular risk: combined post hoc analysis of the SUSTAIN and PIONEER trials
Source: Cardiovasc Diabetol. 2020 Sep 30;19:156. doi: 10.1186/s12933-020-01106-4 (PMC7526237; doi:10.1186/s12933-020-01106-4)
Supplement: Supplementary file 6 — Additional file 6: Figure S1. Forest plots for semaglutide vs comparators across the quartiles of CV risk scores (and its components). *p-value for interaction between treatment and CV risk quartile. Data in parentheses are the risk scores included in each quartile. CI, confidence interval; CV cardiovascular; HR, hazard ratio; MACE, major adverse cardiovascular events; MI, myocardial infarction; Q, quartile. [file 12933_2020_1106_MOESM6_ESM.docx]

**Supplementary Appendix Figure S1.** Forest plots for semaglutide vs comparators across the quartiles of CV risk scores (and its components)


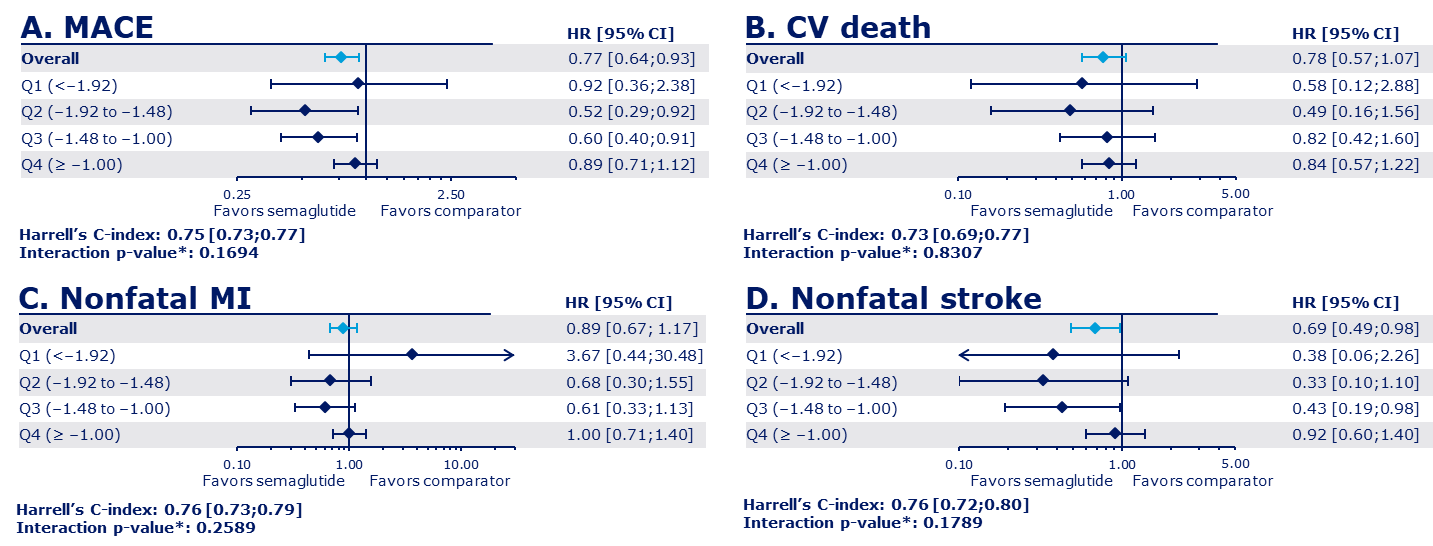


*p-value for interaction between treatment and CV risk quantile. Data in parentheses are the risk scores included in each quartile. CI, confidence interval; CV cardiovascular; HR, hazard ratio; MACE, major adverse cardiovascular events; MI, myocardial infarction; Q, quartile.
