# Supplementary material for: Effects of semaglutide on risk of cardiovascular events across a continuum of cardiovascular risk: combined post hoc analysis of the SUSTAIN and PIONEER trials
Source: Cardiovasc Diabetol. 2020 Sep 30;19:156. doi: 10.1186/s12933-020-01106-4 (PMC7526237; doi:10.1186/s12933-020-01106-4)
Supplement: Supplementary file 7 — Additional file 7: Table S6. Subject profiles, representing the four quartiles of baseline CV risk. Examples of real subject profiles were chosen at the 12.5th, 37.5th, 62.5th and 87.5th percentiles of CV risk score distribution. The factors listed are those that were identified, based on data from LEADER, as having a significant effect on CV risk (no other factors were identified as important). BP, blood pressure; bpm, beats per minute; CV, cardiovascular; eGFR, estimated glomerular filtration rate; HbA1c, glycated hemoglobin; LDL-C, low-density lipoprotein cholesterol; MACE, major adverse cardiovascular events; MI, myocardial infarction; NNT, number needed to treat to avoid one MACE during 1 year; NYHA, New York Heart Association. [file 12933_2020_1106_MOESM7_ESM.docx]

**Supplementary Appendix Table S6.** Subject profiles, representing the four quartiles of baseline CV risk

|  | **Quartile 1**  CV risk score: –2.23  NNT: 295 | | **Quartile 2**  CV risk score –1.69  NNT: 194 | | **Quartile 3**  CV risk score –1.25  NNT: 146 | | **Quartile 4**  CV risk score –0.62  NNT: 113 | |
| --- | --- | --- | --- | --- | --- | --- | --- | --- |
| **Subject profile** | **A** | **B** | **C** | **D** | **E** | **F** | **G** | **H** |
| Hazard ratio | 0.49 | 0.49 | 0.57 | 0.57 | 0.64 | 0.64 | 0.76 | 0.76 |
| Age, years | 54 | 52 | 60 | 62 | 57 | 56 | 70 | 77 |
| HbA_1c_, % | 6.9 | 7.8 | 8.6 | 8.6 | 7.9 | 9.1 | 5.8 | 6.5 |
| Smoking status, current/previous/never | Previous | Never | Never | Never | Previous | Never | Previous | Previous |
| LDL-C, mmol/L | 2.6 | 2.1 | 3.7 | 3.4 | 1.8 | 2.3 | 2.1 | 2.2 |
| Pulse rate, bpm | 65 | 68 | 80 | 76 | 63 | 78 | 80 | 66 |
| Systolic BP, mmHg | 116 | 141 | 137 | 140 | 125 | 125 | 155 | 157 |
| Prior ischemic heart disease, yes/no | No | No | No | No | Yes | Yes | No | Yes |
| Prior MI, yes/no | No | No | No | No | Yes | Yes | No | No |
| Prior stroke, yes/no | No | No | No | No | No | No | Yes | No |
| NYHA class | Other/I | Other/I | Other/I | Other/I | Other/I | Other/I | II | Other/I |
| Insulin use, yes/no | No | No | No | No | No | No | No | No |
| eGFR, ml/min/1.73 m^2^ | 104.2 | 100.6 | 84.0 | 87.7 | 92.3 | 103.9 | 94.0 | 45.4 |

Examples of real subject profiles were chosen at the 12.5th, 37.5th, 62.5th and 87.5th percentiles of CV risk score distribution. The factors listed are those that were identified, based on data from LEADER, as having a significant effect on CV risk (no other factors were identified as important).
BP, blood pressure; bpm, beats per minute; CV, cardiovascular; eGFR, estimated glomerular filtration rate; HbA_1c_, glycated hemoglobin;
LDL-C, low-density lipoprotein cholesterol; MACE, major adverse cardiovascular events; MI, myocardial infarction; NNT, number needed to treat to avoid one MACE during 1 year; NYHA, New York Heart Association.
