# Supplementary material for: Effects of semaglutide on risk of cardiovascular events across a continuum of cardiovascular risk: combined post hoc analysis of the SUSTAIN and PIONEER trials
Source: Cardiovasc Diabetol. 2020 Sep 30;19:156. doi: 10.1186/s12933-020-01106-4 (PMC7526237; doi:10.1186/s12933-020-01106-4)
Supplement: Supplementary file 8 — Additional file 8: Figure S2. Absolute yearly risk of MACE with semaglutide vs comparators combined as a function of baseline CV risk for the CVOTs (A) and glycemic efficacy trials (B). Absolute yearly MACE probabilities, estimated using a stratified Cox proportional hazards model including effects of treatment, CV risk score and interaction between both. The x-axis shows the CV risk score derived from subjects’ baseline characteristics in the semaglutide trials. Data on graph cut off at the 5th and 95th percentile of whole dataset. CV, cardiovascular; CVOT, cardiovascular outcomes trial; MACE, major adverse cardiovascular events. [file 12933_2020_1106_MOESM8_ESM.docx]

**Supplementary Appendix Figure S2.** Absolute yearly risk of MACE with semaglutide vs comparators combined as a function of baseline CV risk for the CVOTs (A) and glycemic efficacy trials (B)


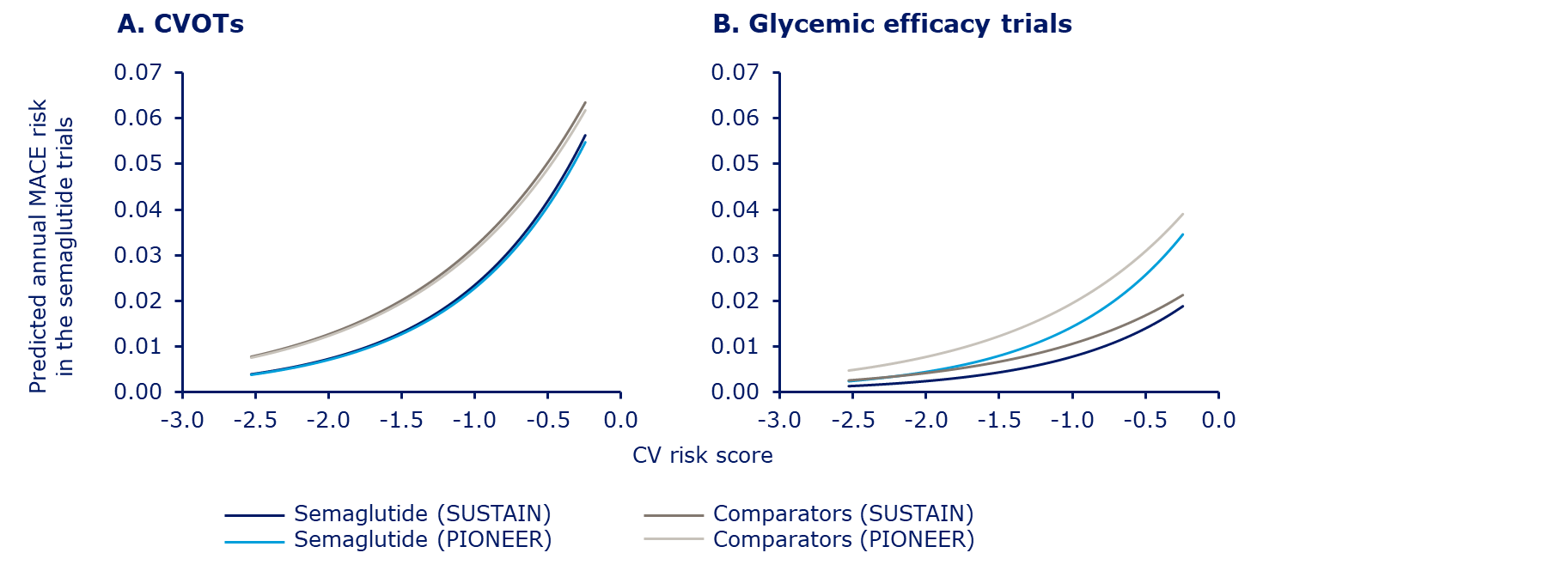


Absolute yearly MACE probabilities, estimated using a stratified Cox proportional hazards model including effects of treatment, CV risk score and interaction between both. The x-axis shows the CV risk score derived from subjects’ baseline characteristics in the semaglutide trials. Data on graph
cut off at the 5th and 95th percentile of whole dataset. CV, cardiovascular; CVOT, cardiovascular outcomes trial; MACE, major adverse cardiovascular events.
