# Supplementary material for: Effects of semaglutide on risk of cardiovascular events across a continuum of cardiovascular risk: combined post hoc analysis of the SUSTAIN and PIONEER trials
Source: Cardiovasc Diabetol. 2020 Sep 30;19:156. doi: 10.1186/s12933-020-01106-4 (PMC7526237; doi:10.1186/s12933-020-01106-4)
Supplement: Supplementary file 9 — Additional file 9: Figure S3. Sensitivity analysis of relative (A) and absolute (B) MACE risk estimates for semaglutide vs placebo (excluding active comparators). Absolute yearly MACE probabilities, estimated using a stratified Cox proportional hazards model including effects of treatment, CV risk score and interaction between both. Includes placebo data from SUSTAIN 1, SUSTAIN 5, PIONEER 1, PIONEER 4, PIONEER 5, PIONEER 6, PIONEER 8 and PIONEER 9. Hazard ratio value of 1.00 is indicated by horizontal dashed line. For panel B the model was without the stratification. The x-axis shows the CV risk score derived from subjects’ baseline characteristics in the semaglutide trials. Data on graph cut off at the 5th and 95th percentile of whole dataset. CI, confidence interval; CV, cardiovascular; CVOT, cardiovascular outcomes trial; HR, hazard ratio; MACE, major adverse cardiovascular events; NNT, number needed to treat to avoid one MACE during 1 year. [file 12933_2020_1106_MOESM9_ESM.docx]

**Supplementary Appendix Figure S3.** Sensitivity analysis of relative (A) and absolute (B) MACE risk estimates for semaglutide vs placebo (excluding active comparators)


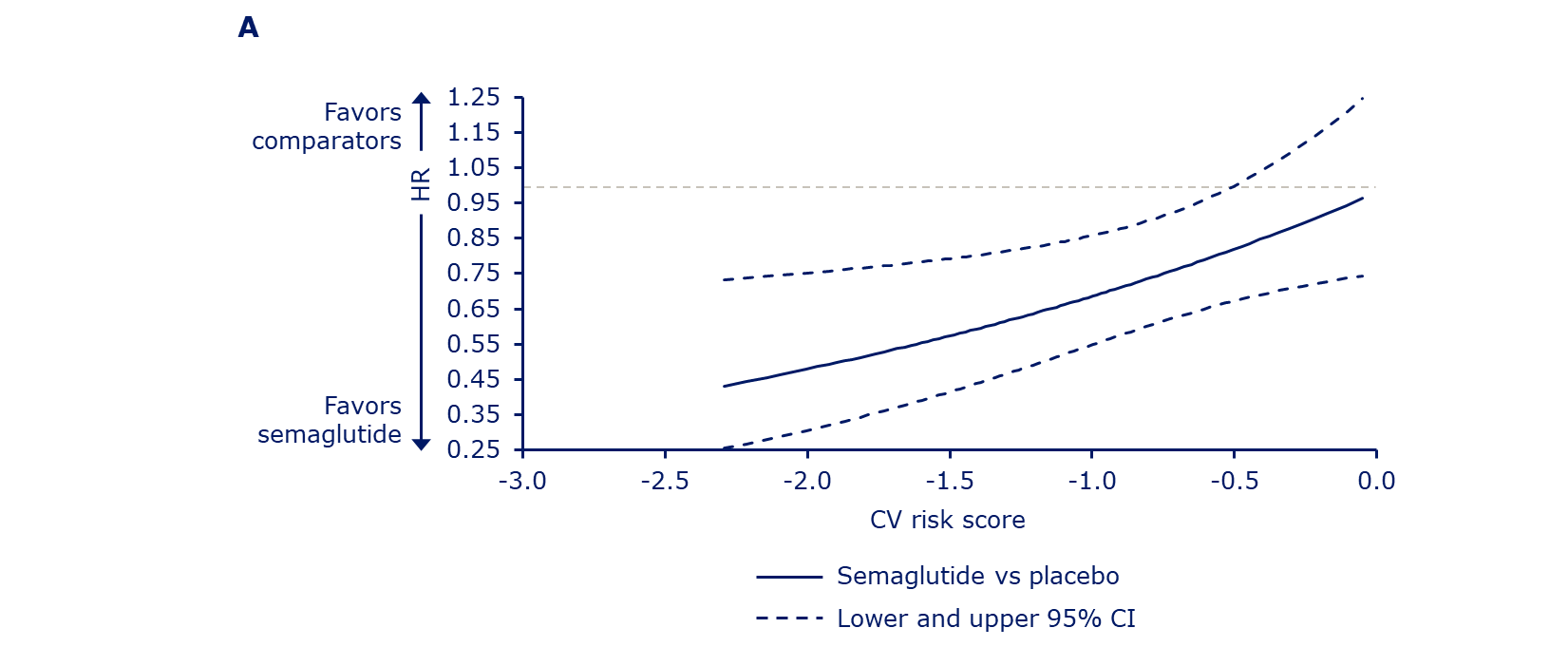


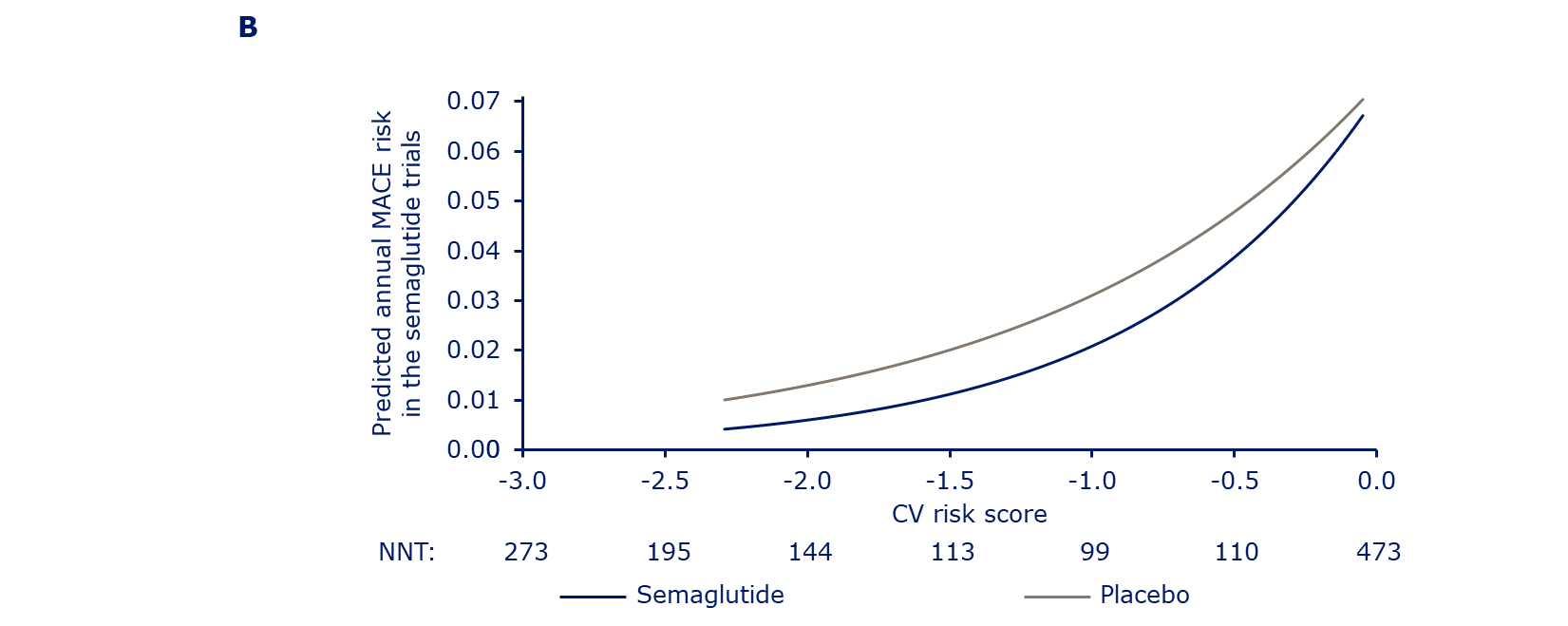


Absolute yearly MACE probabilities, estimated using a stratified Cox proportional hazards model including effects of treatment, CV risk score and interaction between both. Includes placebo data from SUSTAIN 1, SUSTAIN 5, PIONEER 1, PIONEER 4, PIONEER 5, PIONEER 6, PIONEER 8 and PIONEER 9. Hazard ratio value of 1.00 is indicated by horizontal dashed line. For panel B the model was without the stratification. The x-axis shows the CV risk score derived from subjects’ baseline characteristics in the semaglutide trials. Data on graph cut off at the 5th and 95th percentile of whole dataset. CI, confidence interval; CV, cardiovascular; CVOT, cardiovascular outcomes trial; HR, hazard ratio; MACE, major adverse cardiovascular events; NNT, number needed to treat to avoid one MACE during 1 year.
